# Editorial: Novel therapeutic approaches to target drug resistant tumors

**DOI:** 10.3389/fphar.2023.1143981

**Published:** 2023-03-02

**Authors:** Chiara Riganti, Konrad Kowalski, Joanna Kopecka

**Affiliations:** ^1^ Department of Oncology, University of Torino, Torino, Italy; ^2^ Department of Organic Chemistry, Faculty of Chemistry, University of Łódź, Łódź, Poland

**Keywords:** drug resistant tumors, novel therapeutic approaches, drug repurposing, new drugs, nano-delivery systems

To overcome CDR we need to find specific vulnerabilities in cancer cells. One of the weak points of cancers may be dependence on glycolysis as main energy source as shown by Uludag et al. Indeed, targeting the glycolytic enzyme hexokinase-II with methyl jasmonate in glioblastoma cells, know from their high chemoresistence, causes apoptotic/necrotic death and autophagy of cancer cells (Uludag et al.). Alternately, the targeting of selected resistant tumor cell population such as stem cells may be a successful strategy, as shown for multiple myeloma where the overexpression of Bruton’s tyrosine kinase (BTK) in stem cell-like cells is correlated with drug resistance and poor prognosis. A novel small BTK inhibitor, KS151, achieved this goal, because it is unique compared to other BTK inhibitors. Indeed, KS151 can inhibit BTK activities without binding to C481, which is mutated in many patients, causing resistance to BTK inhibitors (Elbezanti et al.).

Another approach to improve cancer therapy outcomes is to synthetize new drugs designed to work at higher efficacy against cancer resistant cells. For instance new compounds containing metals such as organo-ruthenium (II) half-sandwich complexes have been very efficient against primary and highly aggressive metastatic ovarian cancer cells (Guler et al.). Similarly, new-generation synthetic experimental toxoids are effective against paclitaxel resistant NCI/ADR-RES, suppressing tumor growth of NCI/ADR-RES xenografts at low doses (≤3 mg/kg) with no side effects (Seborova et al.). That is of importance because new drugs for treatment of cancers should be maximally safe for patients. This criteria is fulfilled for synthetic H_2_-S-releasing doxorubicin (Sdox). This drug has low affinity for the drug transporter P-glycoprotein (Pgp) therefore it is adapted for the treatment of resistant cells overexpressing Pgp. In addition, this drug is less hepatotoxic, and causes less oxidative damage than doxorubicin, displays better ADME/toxicity profile, different selectivity against cancer targets, along with a greater preclinical efficacy in resistant tumor cells. Hence, Sdox represents a prototype of innovative anthracyclines, worthy of further investigations in clinical settings (Alov et al.).

Another valid solution against CDR is drug repurposing, using drugs with known mechanisms of action and pharmacokinetics, already clinically approved for other diseases. For instance, the combination of metformin, a well-known anti-diabetic drug, and thymoquinone was effective against chronic myeloid leukemia cell lines sensitive and resistant to imatinib therapy. The mechanism of action was linked to increased the levels of cleaved poly (ADP-ribose) polymerase (PARP), decreased the levels of proliferation regulatory proteins, and inhibition of protein kinase B (Akt) and NF-κB signalling (Glamoclija et al.). Similarly, itraconazole, an oral antifungal drug, can efficiently re-sensitize docetaxel resistant prostate and breast cancer cells to treatment. This effect is probably dependent on the inhibition of Pgp, as indicated by molecular modelling studies (Lima et al.).

Finally, to improve therapy outcome the drugs should be specifically targeted to tumors using nano-delivery systems with the ability to overcome drug resistance mechanisms. Unfortunately, nano-carriers have still some problematic Research Topic, including complex preparation processes, low drug-loading capacity, relatively narrow targeting mechanism. All these Research Topic must be solved in order to effectively defeat drug resistant cancers (Zhang et al.).

Summing up there is still a lot to do in the field of CDR to develop new drugs bypassing the most common mechanisms of resistance occurring in tumors, at the diagnosis or after the frontline treatments. Drugs must be effective but also relatively safe for the patients with good toxicity profile and limited side effects. In this perspective, drugs carried by nanocarriers that specifically target tumors may represent a step forward.

